# Consumers’ Corporate Social Responsibility Perception and Anti-Consumer Awareness: Roles of Compassion and Corporate Social Responsibility Authenticity in South Korea

**DOI:** 10.3390/bs13080622

**Published:** 2023-07-26

**Authors:** Sung-Hoon Ko, Ji-Young Kim, Yongjun Choi

**Affiliations:** 1 Graduate School of Education, Kyonggi University, Suwon 16227, Republic of Korea; baumhoffnung@kyonggi.ac.kr; 2 College of Business Administration, Hankuk University of Foreign Studies, Seoul 02450, Republic of Korea; 3 College of Business Administration, Hongik University, Seoul 04066, Republic of Korea; yongjun.choi@hongik.ac.kr

**Keywords:** corporate social responsibility (CSR), CSR perception, compassion, CSR authenticity, anti-consumer awareness

## Abstract

This study examines the effect of consumers’ perceptions of corporate social responsibility (CSR) on their anti-consumer awareness. Specifically, this study aims to uncover the mechanisms through which the consumer’s CSR perception relates to their formation of anti-consumer awareness. A survey design was adopted to test this study’s hypotheses. We collected data from 310 consumers in South Korea and used path analysis and bootstrapping to test the hypotheses. Our results showed that consumers’ CSR perception is negatively related to their anti-consumer awareness. Notably, consumers’ perception of CSR activities is positively related to their perceptions of compassion toward organizations, which, in turn, is also positively associated with their perception of CSR authenticity. Furthermore, compassion and CSR authenticity serially mediate the negative relationship between CSR perception and anti-consumer awareness. Our findings shed light on the importance of engaging in CSR activities from consumers’ perspectives. Specifically, our findings suggest that organizations need to proactively engage in CSR activities with authenticity to maintain and even further their reputation among consumers. Furthermore, by demonstrating the psychological processes of how CSR activities translate into consumers’ attitudes toward the organizations, our study provides fruitful avenues for future research.

## 1. Introduction

Active participation in social contribution activities is crucial for the survival and development of a company [1]. In particular, consumers, as an important company stakeholder who can reward or punish a specific company through actions like purchasing, promote the fulfillment of corporate social responsibility (CSR), which refers to the way that corporations show their concerns and commitment to society [2], more than ever by expecting the company to fulfill its social responsibility and by showing a favorable attitude toward companies that faithfully fulfill their social responsibility [3,4]. Furthermore, a company’s CSR activities can turn a negative attitude that consumers may hold toward the company into a positive one, even when the company is confronted with malicious rumors or adverse events [5]. Therefore, companies must understand how consumers’ perceptions of CSR activities affect their perceptions of these companies.

Past studies have focused on the positive roles of CSR activities, such as increasing consumers’ positive attitudes (e.g., loyalty) towards organizations and purchase intention [3,6,7]. However, the possibility that consumers may question the authenticity of CSR activities (i.e., the degree of an individual’s belief in the authenticity of CSR activities carried out by a company) cannot be ruled out. Therefore, consumers might interpret CSR activities as instrumental means to build a favorable corporate image. Hence, it is important to explore the roles played by CSR authenticity [8] in explaining the mechanisms through which consumers’ CSR perceptions shape their attitudes towards companies.

This study examines the relationship between consumers’ CSR perception and anti-consumer awareness, which refers to a consumer’s perception that a company engages in unjust business practices in ethical or legal terms [9]. Consumers’ perception of CSR activities is important in consumer behavior because it affects their purchase intention as well as attitudes toward organizations [10,11,12]. In other words, consumers’ negative perception of CSR activities can be harmful for organizations. Specifically, anti-consumer awareness can be detrimental for organizations because consumers holding negative perceptions of an organization (i.e., anti-consumer awareness) can pertain to the loss of organizational legitimacy, which is one of the key resources for organizational sustainability [13,14,15].

In addition, we explore the roles of CSR authenticity and compassion in the relationship between consumers’ CSR perception and anti-consumer awareness. Compassion means “an interpersonal process involving the noticing, feeling, sensemaking, and acting that alleviates the suffering of another person” (p. 277) [16]. In other words, compassion is a “… feeling that arises in witnessing another’s suffering which motivates a subsequent desire to help” (p. 352) [17]. In our study context, consumers can also experience compassion toward companies. That is, consumers can experience an emotional state desiring to assist the company while enduring the pain of the company [18]. Past studies on compassion in organizational contexts demonstrate its positive effects on work [16]. For example, regarding the relationship between compassion and CSR authenticity, Ko and Choi [19] showed that there is a positive relationship between the experiences of compassion at work and perceived CSR authenticity. However, our knowledge of the roles of compassion from the consumer’s perspective is still nascent. Fortunately, a few studies have attempted to explore the roles of compassion from the consumer’s perspective. For example, Bae et al. [20] showed that compassion mediates the positive relationship between CSR perception and purchase intention for online game items. Meyer et al. [18] demonstrated that when companies go through negative events for which they are fully responsible, consumers experience compassion for the company, which, in turn, increases their purchasing behaviors. In addition, Ko et al. [21] demonstrated that the compassion experienced by customers using low-cost airlines increases consumers positive attitudes towards the brand. Hence, grounded on past findings, we expect that consumers’ CSR perception helps them form compassion toward organizations, which, in turn, allows them to perceive the authenticity of CSR activities. Notably, even when companies face unfavorable situations (e.g., when suffering from rumors or scandals), it is likely that consumers who perceive CSR authenticity still hold positive attitudes toward organizations. Thus, we expect that they are likely to show less anti-consumer awareness.

This study contributes to the current literature by empirically examining the effect of CSR perception from the consumer’s perspective. Compassion, which is one form of moral emotions, has rarely been explored in the consumer behavior literature, although, as part of human nature, it is pertinent to consumer behaviors [17,18]. Unlike Meyer et al. [18], who focused on the effect of consumer compassion in negative events, we examine not only the outcome but also the antecedent of consumer compassion. More specifically, we examine the serial mediation effect of compassion and CSR authenticity in the relationship between CSR perception and anti-consumer awareness. By so doing, our results illuminate the important roles played by CSR activities from the consumer’s perspective, providing fruitful areas of research for future studies. Essentially, our findings provide companies with implications they need to authentically and proactively engage in CSR activities to help consumers form and hold positive attitudes toward them.

## 2. Theoretical Background and Hypothesis Development

Past studies focusing on the effect of CSR perception in organizational contexts showed that employees with high CSR perception are more likely to experience compassion at work, which, in turn, increases their positive attitudes and behaviors at work [21,22,23,24]. In a similar vein, drawing upon social identity theory, Peterson [25] demonstrated that CSR perception helps employees perceive high levels of self-esteem by experiencing positive emotions at work.

By extending past studies, this study proposes that customers with high CSR perceptions towards certain companies are more likely to display compassionate reactions toward these companies. CSR activities can induce positive emotions in consumers and reduce various risk factors that companies can experience by helping consumers build trust in companies [26]. In particular, CSR perception leads to consumers having positive attitudes and behavioral intentions [3]. Therefore, it is possible to predict that when a company with a favorable CSR perception experiences a difficult problem, consumers will display a compassionate response, as if sharing in the company’s suffering, showing an active intention to purchase more of the company’s product [18,27]. Thus, we hypothesize the following:

**H1.**  *CSR perception is positively related to compassion.*

Prior studies have defined compassion as empathizing with the recognition and suffering of others’ pain and showing affection and kindness in various ways for alleviating such pain [28,29,30]. Solomon [31] argued that compassion could occur in human relationships; that is, emotional elements such as a bond between oneself and others or interest in others could have a deep relationship with compassion, and compassion is shown through sadness as a response to the pain experienced when others are in a difficult situation. 

Past studies on compassion within organizational settings have demonstrated its positive effects on work. For example, while demonstrating that compassion triggers positive emotions, Lilius et al. [27] suggested that compassionate actions could strengthen authenticity. Compassion can be a relational process that pays attention to and empathizes with the suffering of others and induces authentic behavior that could alleviate this suffering [29]. Miller [32] regarded compassion as emotional work with authenticity that occurs naturally in the heart, not as an emotion generated by artificial effort. In other words, the compassionate response that consumers demonstrate when a company performing CSR activities undergoes pain can further strengthen the consumer–company bond [33] and have consumers perceive the company’s CSR activities as being authentic. Thus, we hypothesize the following:

**H2.**  *Compassion is positively related to CSR authenticity.*

CSR authenticity not only means the consumer’s actual trust in CSR activities [34] but is also an important factor influencing the evaluation of CSR activities [35,36]. In particular, authenticity is a critical factor for stakeholders in evaluating CSR activities [35]. For example, customers could easily lose their trust toward companies lacking authenticity in their CSR activities [37]. Hence, CSR authenticity could serve as an important factor affecting customers’ attitudes and behaviors towards companies. Likewise, past studies emphasize the importance of brand authenticity in developing and maintaining the positive reputation of companies [38].

When consumers feel a sense of authenticity in a brand, they perceive the product or service they consume as differentiated, thus having a more positive evaluation of the brand [39]. Therefore, authenticity can prove an important factor in evaluating various corporate activities [35].

When consumers feel a sense of authenticity regarding a company’s CSR activities, distrust in the company is reduced. Authenticity can be viewed as trust in an actual object [34], and this trust induces “trust transfer” in consumers to make a qualitative evaluation of the product [40]. Therefore, actions without authenticity are less likely to elicit positive responses than actions with authenticity, even if the action itself is not criticized [41]. Therefore, the authenticity of CSR activities can reduce a negative perception of the company’s activities among consumers and even lead to a positive evaluation of the company. Hence, we hypothesize the following:

**H3.**  *CSR authenticity is positively related to anti-consumer awareness.*

Perceptions of CSR activities can have positive effects (e.g., improving corporate image and strengthening competitiveness) [3,42,43,44]. In particular, consumers’ awareness of CSR through communications that foster agreement between stakeholders’ expectations about corporate responsibility and CSR agendas [45] improves consumers’ emotional immersion in the company by increasing their awareness of social problems and interest in social causes [46].

Consumers positively evaluate the image of a company that engages in CSR activities, leading to a high intention to purchase the company’s product [47]. Prior studies reveal that when a company experiences a crisis, the corporate reputation or image formed through CSR activities can help protect the company against negative factors [5,48]. Thus, it can be predicted that consumers’ negative perceptions of companies that authentically engage in CSR activities will decrease.

**H4.**  *CSR perception is negatively related to anti-consumer awareness.*

The positive relationship between CSR activities and organizational performance is well established in the field [49,50,51]. CSR activities encourage consumers to form positive attitudes toward and maintain their interest in a company. However, the direct relationship between CSR perception and consumer response is unclear and debated in most studies [52,53]. Therefore, the influence of the companies that directly interact with consumers on CSR activities is not always clear and may vary depending on other factors [54,55].

Consumers view CSR activities as positive virtues. Such virtuous actions elicit positive emotions such as love, compassion, and passion [56]. For example, employees who have experienced virtuous actions, like social responsibility activities, will experience compassion. They will display compassion for each other and gratitude for the organization based on the positive emotions formed from experiencing compassion [57]. Such behavior accompanies an emotion developed through feeling and experiencing the pain of others as well as one’s own pain [58], and this kind of empathy can reflect an emotional state by expressing a warm heart or goodwill and kindness [59]. Therefore, it can be predicted that consumers who have experienced compassionate responses can accept CSR activities as being more authentic.

Authenticity is ‘intrinsically truthful’, reflecting whether the other party’s specific behavior is recognized as an act that comes from a heart that sincerely cares for the other party rather than a sense of duty or responsibility [8]. CSR authenticity, a concept that applies authenticity like the former to CSR activities, means that CSR activities go beyond legal requirements, and the company’s beliefs for society are expressed in more genuine actions [60]. Therefore, when social responsibility activities are recognized as pure and sincere corporate activities, they positively affect corporate attitudes [42,61], and CSR authenticity can serve as a critical factor in the evaluation (e.g., positive perception about the activity) [35]. Therefore, as consumers who recognize CSR activities experience compassion, they can feel CSR authenticity and have less anti-consumer awareness. Therefore, we hypothesize that there is a double mediation effect of compassion and CSR authenticity on the negative relationship between CSR perception and anti-consumer awareness.

**H5.**  *Compassion and CSR authenticity serially mediate the negative relationship between CSR perception and anti-consumer awareness.*

**Figure 1:**
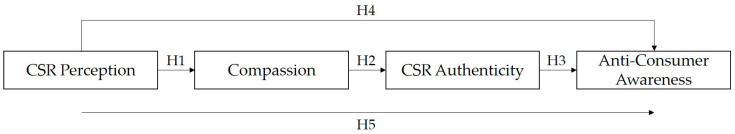
Research model.

Figure 1 depicts our research model.

## 3. Methods

### 3.1. Study Participants and Procedure

A professional research company with more than 1.5 million panelists was employed to survey general consumers who could respond to a questionnaire about CSR activities. The survey was conducted online in March 2021. The professional research company restricted the samples to only consumers who had experiences of purchasing products or services from large domestic companies. Participants were asked to voluntarily participate in this study, and compensation was provided in the form of a gift card worth USD 5.

The survey questionnaires were distributed to 320 people in their 20s and older who live in metropolitan areas. A total of 310 questionnaires were used for statistical data analysis, with 10 excluded due to a centralization tendency or incomplete answers. The respondents consisted of 157 men (50.6%) and 153 women (49.9%), with 80 respondents (25.8%) each in their 20s, 30s, and 40s, and 48 respondents being high school graduates (15.5%), 53 two-year community college graduates (17.1%), and 164 four-year college graduates (52.9%).

To test our research model, first, we conducted frequency analysis, correlation analysis, descriptive statistics, and reliability analysis using SPSS 23.0. In addition, we used AMOS 24.0 for CFA analysis, CMV analysis, and path analysis. Lastly, we used bootstrapping for testing our dual mediation hypothesis.

### 3.2. Measures

The research model used in this study is composed of four constructs: CSR perception, compassion, CSR authenticity, and anti-consumer awareness. A five-point Likert scale ranging from ‘strongly disagree’ (coded as ‘1’) through ‘strongly agree’ (coded as ‘5’) was used to measure each question item. Table 1 summarizes the measurements we used in this study.

## 4. Results

### 4.1. Confirmatory Factor Analysis

After removing one item for CSR perception (due to its low factor loading), we conducted confirmatory factor analysis (CFA) using AMOS 24.0 to determine the goodness of fit of our research model. The results are as follows: χ^2^ = 647.987 (df = 347, *p* = 0.000), CFI = 0.959, TLI = 0.952, NFI = 0.917, RMSEA = 0.053, RMR = 0.033, indicating that the overall goodness of fit exceeds the fit criteria. Next, the internal consistency of latent variables was determined with the coefficient of Cronbach’s alpha. In this regard, all coefficients were greater than 0.8, demonstrating no problems with the internal consistency since the coefficients were greater than 0.7 [65]. Furthermore, the average variance extracted (AVE) values were all greater than 0.6, indicating that they meet the traditional criteria.

### 4.2. Hypotheses Testing

This study used the Pearson correlation coefficient to examine multicollinearity. The results show the coefficients between −0.314 and 0.585 (*p* < 0.01), demonstrating that multicollinearity was not a serious issue in this study. This study also examined convergent validity to assess the correlation between observed variables and discriminant validity, examining the correlation between latent variables [66].

As shown in Table 2, the values of the standardized lambda, AVE, and conceptual reliability of all measurement items satisfied the conventional criteria. Therefore, it can be concluded that the measurement items for the latent variables used in this study have secured convergent and discriminant validity.

We used path analysis to test our hypotheses. Path analysis is one form of structural equational modeling (SEM) applied when all the variables can be quantified with no latent variables [67,68,69]. Accordingly, AMOS 24.0 was used to test the hypotheses, and the results are as follows. The path coefficient between CSR perception and compassion was β = 0.962 (t = 15.399, *p* < 0.001), supporting Hypothesis 1; the path coefficient between compassion and CSR authenticity was β = 0.715 (t = 19.297, *p* < 0.001), supporting Hypothesis 2; the path coefficient between CSR authenticity and anti-consumer awareness was β = −0.175 (t = −2.377, *p* < 0.01), supporting Hypothesis 3; and the path coefficient between CSR perception and anti-consumer awareness was β= −0.123 (t = −1.970, *p* < 0.01), supporting Hypothesis 4. The path analysis results are presented in Table 3.

A bootstrapping estimate was used to test Hypothesis 5, regarding the dual mediation effect of compassion and CSR authenticity [70]. As shown in Table 4, the bootstrap estimate of the indirect effect between CSR perception and anti-consumer awareness was found to be significant (LL95CI = −0.1293, UL95CI = −0.0011), supporting Hypothesis 5 that compassion and CSR authenticity serially mediate the negative relationship between CSR perception and anti-consumer awareness.

### 4.3. Common Method Variance

This study used self-reported data, leaving room for possible common method bias that may affect the validity of the study’s results [71,72]. The common method bias was examined by controlling for the effects of a latent methods factor. This technique examines changes in the significance of the structural parameters between the cases with and without the latent methods factor in the model (e.g., the χ^2^ difference according to the change in degrees of freedom, the change in the model fit, and the estimate and significance of path coefficients) [71,73]. If the changes are significant, a common method bias exists. The results are presented in Table 5.

The model fit indices were χ^2^ = 647.987 (df = 347, *p* = 0.000), CFI = 0.959, TLI = 0.952, NFI = 0.917, RMSEA = 0.053, RMR = 0.033 before controlling for the effects of a latent methods factor and χ^2^ = 567.557 (df = 318, *p* = 0.000), CFI = 0.966, TLI = 0.957, NFI = 0.927, RMSEA = 0.050, RMR = 0.052 after controlling. The difference in the Δχ^2^ value (80.41) according to the change in degrees of freedom (df = 29) between the cases with and without controlling for the latent methods factor turned out to be insignificant (*p* > 0.05), indicating a potentially low common method bias. Furthermore, the absolute value of the λ-CMV, which is the difference between the λ values before and after controlling for the latent methods factor, did not exceed 0.2, also indicating a low possibility of a common method bias in this research model [71,74]. A model that does not control for the effect of the latent methods factor was used for hypothesis testing [73].

## 5. Discussion

### 5.1. General Discussion

The present study aimed to investigate the relationship between consumers’ perception of CSR activities and anti-consumer awareness, while exploring the mediating roles of compassion and CSR authenticity. Our results indicate that consumers’ perception of CSR is negatively related to their anti-consumer awareness via the increases in their experiences of compassion followed by their perception of CSR authenticity, providing valuable insights into the roles of CSR perception in the formation of consumers’ attitudes toward companies.

Overall, our findings are consistent with the past studies on CSR demonstrating the positive effects of CSR activities from consumers’ perspectives [3,6,7,10,11,12]. First, consistent with previous studies, our results demonstrate a positive relationship between CSR perception and compassion. Consumers who perceive a company as actively engaged in CSR activities are more likely to experience compassion towards that company [18]. This finding aligns with the notion that CSR activities can evoke positive emotions in consumers, leading to increased empathy and a desire to support the company [19,25]. Second, our study reveals a positive relationship between compassion and CSR authenticity. Consumers who feel compassion towards a company are more likely to perceive its CSR activities as genuine and sincere [27,32], suggesting that compassionate responses from consumers can reinforce the authenticity of CSR initiatives, creating a stronger bond between consumers and the company. Third, our findings indicate a negative relationship between CSR authenticity and anti-consumer awareness. Consumers who perceive a company’s CSR activities as authentic are less likely to exhibit anti-consumer awareness, suggesting that authenticity plays a crucial role in the formation of consumers’ attitudes toward the company. Fourth, our study demonstrates a negative relationship between CSR perception and anti-consumer awareness. That is, consumers who hold positive perceptions of a company’s CSR activities are less likely to exhibit anti-consumer awareness, emphasizing the importance of CSR perception in shaping consumers’ attitudes towards companies. Finally, our study investigates the serial mediation effect of compassion and CSR authenticity on the relationship between CSR perception and anti-consumer awareness. The results support this hypothesis, indicating that compassion and CSR authenticity sequentially mediate the negative relationship between CSR perception and anti-consumer awareness. That is, consumers who perceive CSR activities positively are more likely to experience compassion, which, in turn, leads to perceiving the CSR activities as more authentic. Accordingly, this perception of CSR authenticity reduces their anti-consumer awareness.

### 5.2. Theoretical and Practical Implications

The theoretical implications of this study are as follows: first, this study revealed the relationship between consumers’ perceptions of CSR and anti-consumer awareness. CSR is receiving increasing attention in consumer behavior (and, more broadly, in marketing) as it treats consumers as one of the stakeholders of an organization [75]. From consumers’ perspectives, past studies on CSR in marketing have mainly focused on its positive effects [76], such as consumers’ positive evaluation of companies and even their products and services [77,78]. Extending the previous studies, our study focused on the roles of CSR in diminishing consumers’ negative attitudes. Specifically, our findings demonstrated the negative relationship between consumers’ perception of CSR and anti-consumer awareness. Therefore, our findings contribute to the current literature with insights that CSR activities are not only beneficial for energizing consumers’ positive attitudes but also lessen their negative attitudes towards companies engaging in CSR activities.

Second, this study empirically examined a dual mediation effect of compassion and CSR authenticity on the negative relationship between CSR perception and anti-consumer awareness, providing another theoretical implication. Consumers’ experiences of compassion can not only help them develop positive attitudes towards companies but also trigger their behaviors to help companies even when they experience negative events [18]. Similarly, our findings showed that consumers perceiving CSR activities are more likely to experience compassion and, thus, conclude that CSR activities are authentic which, in turn, lessens their anti-consumer awareness. It is noteworthy that, unlike previous studies examining the relationship between CSR perception and CSR authenticity [79,80], we further showed the roles of compassion in this link. Therefore, our findings provide a psychological mechanism through which consumers’ CSR perception plays out in the context of marketing. In addition, extending the current literature on compassion in consumer behavior [18,20,21], this study provides additional evidence highlighting the roles of compassion, which enriches our understanding of the roles of compassion in consumer behavior.

Our findings also provide organizations with practical implications. First, this study found that CSR perception can be an important antecedent factor in reducing anti-consumer awareness. Unlike previous studies focused on perceptions within the organization, this study showed how CSR activities induce positive psychological emotions in consumers and reduce anti-consumer awareness about companies, suggesting a practical implication for preventing negative perceptions of companies. 

Second, this study shows that when consumers perceive compassion for a company in crisis and CSR authenticity about a company engaging in CSR activities, their antipathy toward the company (i.e., anti-consumer awareness) is reduced. In the case of a company that has experienced an adverse event, often, the negative perception cannot be overcome despite the company’s various efforts, like making an apology statement to consumers. However, in the case of a company that has been consistently carrying out CSR activities, the CSR activities induce consumers’ compassionate acts to feel and experience the others’ pain (i.e., the company in crisis in this case), leading consumers to recognize the authenticity of the company’s CSR activities. A practical implication here is that having consumers recognize CSR activities can induce their positive responses, such as increased compassion and CSR authenticity and reduced anti-consumer awareness.

Third, this study shows that when a company engaged in CSR activities suffers from a crisis, like worsening business difficulties or decreased sales, consumers perceiving the authenticity of CSR activities have less anti-consumer awareness about the company. A practical implication from this finding is apparent: companies that have not received empathy from consumers while conducting inauthentic CSR can gain empathy from these consumers through authentic CSR activities in the future.

### 5.3. Limitations and Future Research Directions

Our study is not without limitations. First, it may be difficult to generalize the findings from this study to all companies and consumers since the study involved only Korean companies and consumers. In particular, the survey respondents were consumers living in specific areas of Seoul, Gyeonggi, and Incheon in South Korea. Residence may be divided to maintain one’s socio-economic status [81]. Therefore, future research warrants consideration of possible significant differences in the overall region. It is necessary to research various countries and consumers or carry out comparative studies on companies and consumers between the Republic of Korea and other countries.

Second, the outcome variable affected by consumers’ CRS perception, compassion, and CSR authenticity is limited to anti-consumer awareness in this study. However, various consumer behavior-related variables such as repurchase intention, forgiveness, and regret can be considered outcome variables. Therefore, future research is warranted to include different outcome variables and empirically test their relationships with various mediating variables.

Third, while our research model offers a valuable framework for examining the relationships between consumers’ perception of CSR, compassion, CSR authenticity, and anti-consumer awareness, we acknowledge that these relationships may be influenced by additional factors and non-linear dynamics. In other words, this simplification may limit our understanding of the full complexity of the phenomena. Nevertheless, it is important to note that this linear representation also facilitates a better understanding of the phenomena by presenting a simplified framework for investigating the relationships between our study variables. Hence, future research could benefit from exploring alternative modeling approaches, such as nonlinear models, to gain a deeper understanding of the intricacies and interdependencies among our study variables.

### 5.4. Conclusions

This study empirically examined the relationships between CSR perception, compassion, and CSR authenticity in the formation of anti-consumer awareness. The results reveal a positive relationship between CSR perception and compassion and between compassion and CSR authenticity and a negative relationship between CSR perception and anti-consumer awareness. These results reveal that the experience of compassion through the recognition of CSR activities can be a mechanism to increase the authenticity of CSR activities. Furthermore, the results show that when consumers sense the CSR authenticity of a company, anti-consumer awareness about the company decreases. Therefore, the stronger the CSR authenticity, the higher the positive attitude toward the brand, as shown in prior studies [82]. This study also identified a more concrete mechanism by testing the dual mediation effect of compassion and CSR authenticity on the relationship between CSR perception and anti-consumer awareness.

## Figures and Tables

**Table 1:** Measurements.

Variable	Source	α	Sample Items
CSR perception	Maignan and Ferrell [12]	0.953	- I think the company has a comprehensive code of ethics guidelines. - I think the company is following the standards of professional ethics.
Compassion	Meyer et al. [18] Lilius et al. [27]	0.899	- I want to spend my time helping the company in crisis or difficulty. - I think the company is a system that can maintain a work–life balance I think I have.
CSR authenticity	Price et al. [8] Schaefer and Pettijohn [62] Kim [63]	0.946	- I believe that the company’s social responsibility activities are sincere. - I believe the company’s social responsibility activities seem to genuinely care for members of society.
Anti-consumer awareness	Barksdale and Darden [64]	0.830	- I think large corporations in Korea lack a sense of social responsibility. - I think that large corporations in Korea are conducting unethical management practices, like accounting fraud.

Note: α denotes Cronbach’s alpha.

**Table 2:** Construct means, standard deviations, and correlations.

	Mean	SD	1	2	3	4
1. CSR perception	3.287	0.649	0.844			
2. Compassion	2.613	0.947	0.559 **	0.939		
3. CSR authenticity	3.034	0.916	0.585 **	0.539 **	0.945	
4. Anti-consumer awareness	3.757	0.750	−0.281 **	−0.314 **	−0.340 **	0.844

Note: N = 310; ** *p* < 0.01. The numbers along the diagonal are the square root of the averaged variance extracted (AVE).

**Table 3:** Path analysis results.

Hypothesis	Path	Estimate	S.E.	C.R.	*p*
H1	CSR perception → Compassion	0.962	0.062	15.399	<0.001
H2	Compassion → CSR authenticity	0.715	0.037	19.297	<0.001
H3	CSR authenticity → Anti-consumer awareness	−0.175	0.074	−2.377	<0.01
H4	CSR perception → Anti-consumer awareness	−0.123	0.062	−1.970	<0.01

**Table 4:** Indirect effects for the double-mediation effects.

Hypothesis	Effect	LLCI95%	ULCI95%	BootSE
Total indirect effect	−0.3083	−0.4595	−0.1540	0.0775
CSR perception → Compassion → ACA	−0.1295	−0.2606	−0.0020	0.0673
CSR perception → CSR authenticity → ACA	−0.1160	−0.2345	−0.0020	0.0585
CSR perception → Compassion	−0.0628	−0.1293	−0.0011	0.0322

Note: ACA = anti-consumer awareness. Bootstrap confidence intervals (CIs) were constructed using 5000 resamples.

**Table 5:** Analysis of common method bias.

	χ^2^	df	*p*	χ^2^/df	RMSEA	CFI	NFI	TLI
Measurement Model (M.M.)	647.987	347	<0.001	1.86	0.053	0.959	0.917	0.952
Controlled Model (C.M.)	567.557	318	<0.001	1.78	0.050	0.966	0.927	0.957
Stepwise Analysis	Δχ^2^	Δdf		Accepted Model				
M.M.-C.M.	80.43	29	>0.05	Measurement Model				

## Data Availability

The data presented in this study are available on request from the corresponding author.

## References

[1] Vilanova M., Lozano J.M., Arenas D. (2009). Exploring the nature of the relationship between CSR and competitiveness. J. Bus. Ethics.

[2] Ashrafi M., Adams M., Walker T.R., Magnan G. (2018). How corporate social responsibility can be integrated into corporate sustainability: A theoretical review of their relationships. Int. J. Sust. Dev. World.

[3] Brown T.J., Dacin P.A. (1997). The company and the product: Corporate associations and consumer product responses. J. Mark..

[4] Lerro M., Vecchio R., Caracciolo F., Pascucci S., Cembalo L. (2018). Consumers’ heterogeneous preferences for corporate social responsibility in the food industry. Corp. Soc. Responsib. Environ. Manag..

[5] Dawar N., Pillutla M.M. (2000). Impact of product-harm crises on brand equity: The moderating role of consumer expectations. J. Mark. Res..

[6] Lorge S. (1998). Is cause-related marketing worth it?. Sales Mark. Manag..

[7] MacMillan K., Money K., Downing S., Hillenbrand C. (2005). Reputation in relationships: Measuring experiences, emotion and behavior. Corp. Reput. Rev..

[8] Price L.L., Arnould E.J., Tierney P. (1995). Going to extremes: Managing service encounters and assessing provider performance. J. Mark..

[9] Rayne D., Leckie C., McDonald H. (2020). Productive partnerships? Driving consumer awareness to action in CSR partnerships. J. Bus. Res..

[10] Becker-Olsen K.L., Cudmore B.A., Hill R.P. (2006). The impact of perceived corporate social responsibility on consumer behavior. J. Bus. Res..

[11] Lichtenstein D.R., Drumwright M.E., Braig B.M. (2004). The effect of corporate social responsibility on customer donations to corporate-supported nonprofits. J. Mark..

[12] Maignan I., Ferrell O.C. (2001). Corporate citizenship as a marketing instrument, concepts, evidence & research directions. Eur. J. Mark..

[13] Meyer J.W., Rowan B. (1977). Institutionalized organizations: Formal structure as myth and ceremony. Am. J. Sociol..

[14] Suchman M.C. (1995). Managing legitimacy: Strategic and institutional approaches. Acad. Manag. Rev..

[15] Scott W.R., Ruef M., Mendel P.J., Caronna C.A. (2000). Institutional Change and Healthcare Organizations: From Professional Dominance to Managed Care.

[16] Dutton J.E., Workman K.M., Hardin A.E. (2014). Compassion at work. Annu. Rev. Organ. Psych..

[17] Goetz J.L., Keltner D., Simon-Thomas E. (2010). Compassion: An evolutionary analysis and empirical review. Psychol. Bull..

[18] Meyer F., Huber F., Huber S. (2019). The suffering company: Consumer compassion towards companies exposed to negative events. Psychol. Market..

[19] Ko S., Choi Y. (2020). The effects of compassion experienced by SME employees on affective commitment: Double-mediation of authenticity and positive emotion. Manag. Sci. Lett..

[20] Bae J., Park H.H., Koo D.M. (2019). Perceived CSR initiatives and intention to purchase game items: The motivational mechanism of self-esteem and compassion. Internet Res..

[21] Ko S.H., Choi Y., Kim J. (2021). Customers’ experiences of compassion and brand attitude: Evidence from low-cost carriers. Front. Psychol..

[22] Ko S.H., Moon T.W. (2013). The effect of CSR perception within organizations on organizational commitment—Focusing on the mediation effect of compassion. Manag. Inf. Syst. Rev..

[23] Moon T.W., Hur W.M., Ko S.H., Kim J.W., Yoon S.W. (2014). Bridging corporate social responsibility and compassion at work: Relations to organizational justice and affective organizational commitment. Career Dev. Int..

[24] Nazir O., Islam J.U. (2020). Effect of CSR activities on meaningfulness, compassion, and employee engagement: A sense-making theoretical approach. Int. J. Hosp. Manag..

[25] Peterson D.K. (2004). The relationship between perceptions of corporate citizenship and organizational commitment. Bus. Soc..

[26] Nicolau J.L. (2008). Corporate social responsibility: Worth-creating activities. Ann. Tourism Res..

[27] Lilius J.M., Worline M.C., Maitlis S., Kanov J., Dutton J.E., Frost P. (2008). The contours and consequences of compassion at work. J. Organ. Behav..

[28] Dutton J.E., Worline M.C., Frost P.J., Lilius J.M. (2006). Explaining compassion organizing. Admin. Sci. Quart..

[29] Kanov J.M., Maitlis S., Worline M.C., Dutton J.E., Frost P.J., Lilius J.M. (2004). Compassion in organizational life. Am. Behav. Sci..

[30] Kornfield J. (1993). A Path with Heart.

[31] Solomon R.C. (1998). The moral psychology of business: Care and compassion in the corporation. Bus. Ethics Q..

[32] Miller K.I. (2007). Compassionate communication in the workplace: Exploring processes of noticing, connecting, and responding. J. Appl. Commun. Res..

[33] Frost P.J., Dutton J.E., Worline M.C., Wilson A. (2000). Narratives of compassion in organizations. Emot. Organ..

[34] Beverland M. (2006). The ‘real thing’: Branding authenticity in the luxury wine trade. J. Bus. Res..

[35] Beckman T., Colwell A., Cunningham P.H. (2009). The emergence of corporate social responsibility in Chile: The importance of authenticity and social networks. J. Bus. Ethics.

[36] O’Connor A., Shumate M., Meister M. (2008). Walk the line: Active moms define corporate social responsibility. Pub. Relat. Rev..

[37] Leigh T.W., Peters C., Shelton J. (2006). The consumer quest for authenticity, the multiplicity of meanings within the M.G. subculture of consumption. J. Acad. Market. Sci..

[38] Greyser S.A. (2009). Corporate brand reputation and brand crisis management. Manag. Decis..

[39] Morhart F., Malär L., Guèvremont A., Girardin F., Grohmann B. (2015). Brand authenticity: An integrative framework and measurement scale. J. Consum. Psychol..

[40] Stewart K.J. (2003). Trust transfer on the world wide web. Organ. Sci..

[41] Ekman P. (2003). Emotions Revealed: Recognizing Faces and Feelings to Improve Communication and Emotional Life.

[42] Forehand M.R., Grier S. (2003). When is honesty the best policy? The effect of stated company intent on consumer skepticism. J. Consum. Psychol..

[43] Porter M.E., Kramer M.R. (2002). The competitive advantage of corporate philanthropy. Harvard Bus. Rev..

[44] Sen S., Bhattacharta C.B. (2001). Does doing good always lead to doing better? Consumer reactions to corporate social responsibility. J. Mark. Res..

[45] Dawkins J. (2005). Corporate responsibility: The communication challenge. J. Commun. Manag..

[46] Morsing M., Schultz M. (2006). Corporate social responsibility communication: Stakeholder information, response and involvement strategies. Bus. Ethics.

[47] David P., Kline S., Dai Y. (2005). Corporate social responsibility, practices, corporate identity, and purchase intention: A dual-process model. J. Public Relat. Res..

[48] Klein J., Dawar N. (2004). Corporate social responsibility and consumers’ attributions and brand evaluations in a product-harm crisis. Int. J. Res. Mark..

[49] Campbell J.L. (2007). Why would corporations behave in socially responsible ways? An institutional theory of corporate social responsibility. Acad. Manag. Rev..

[50] Orlitzky M., Schmidt F.L., Rynes S.L. (2003). Corporate social and financial performance: A meta-analysis. Organ. Sci..

[51] Waddock S.A., Graves S.B. (1997). The corporate social performance-financial performance link. Strateg. Manag. J..

[52] Gürlek M., Düzgün E., Uygur S.M. (2017). How does corporate social responsibility create customer loyalty? The role of corporate image. Soc. Responsib. J..

[53] He H., Li Y. (2011). CSR and service brand: The mediating effect of brand identification and moderating effect of service quality. J. Bus. Ethics.

[54] Castaldo S., Perrini F., Misani N., Tencati A. (2009). The missing link between corporate social responsibility and consumer trust: The case of fair trade products. J. Bus. Ethics.

[55] Liu M.T., Wong I.A., Shi G., Chu R., Brock J.L. (2014). The impact of corporate social responsibility (CSR) performance and perceived brand quality on customer-based brand preference. J. Serv. Mark..

[56] Cameron K.S., Bright D., Caza A. (2004). Exploring the relationships between organizational virtuousness and performance. Am. Behav. Sci..

[57] Rego A., Ribeiro N., Cunha M. (2010). Perceptions of organizational virtuousness and happiness as predictors of organizational citizenship behaviors. J. Bus. Ethics.

[58] Lilius J.M., Kanov J.M., Dutton J.E., Worline M.C., Maitlis S., Cameron K.S., Spreitzer G. (2011). Compassion revealed: What we know about compassion at work (and where we need to know more). Handbook of Positive Organizational Scholarship.

[59] Kahn W.A. (1993). Caring for the caregivers: Patterns of organizational caregiving. Admin. Sci. Quart..

[60] Alhouti S., Johnson C.M., Holloway B.B. (2016). Corporate social responsibility authenticity: Investigating its antecedents and outcome. J. Bus. Res..

[61] Ellen P., Webb D., Mohr L. (2006). Building corporate associations: Consumer attributions for corporate socially responsible programs. J. Acad. Market. Sci..

[62] Schaefer A.D., Pettijohn C.E. (2006). The relevance of authenticity in personal selling: Is genuineness an asset or liability?. J. Mark. Theory Pract..

[63] Kim S.H. (2010). Did Customer truly forgive service failure company? Authenticity of company recovery efforts and the forgiveness process of customer. Korea Bus. Rev..

[64] Barksdale H.C., Darden W.R. (1972). Consumer attitudes toward marketing and consumerism. J. Mark..

[65] Peterson R.A. (1994). A meta-analysis of Cronbach’s coefficient alpha. J. Consum. Res..

[66] Fornell C., Larcker D.F. (1981). Evaluating structural equation models with unobservable variables and measurement error. J. Mark. Res..

[67] Buhi E.R., Goodson P., Neilands T.B. (2007). Structural equation modeling: A primer for health behavior researchers. Am. J. Health Behav..

[68] Gargoum S.A., El-Basyouny K. (2016). Exploring the association between speed and safety: A path analysis approach. Accid. Anal. Prev..

[69] Li Y., Rashid A., Wang H., Hu A., Lin L., Yu C.-P., Chen M., Sun Q. (2018). Contribution of biotic and abiotic factors in the natural attenuation of sulfamethoxazole: A path analysis approach. Sci. Total Environ..

[70] Preacher K.J., Hayes A.F. (2004). SPSS and SAS procedures for estimating indirect effects in simple mediation models. Behav. Res. Meth. Ins. C..

[71] Podsakoff P.M., MacKenzie S.B., Lee J.Y., Podsakoff N.P. (2003). Common method biases in behavioral research: A critical review of the literature and recommended remedies. J. Appl. Psychol..

[72] Podsakoff P.M., MacKenzie S.B., Podsakoff N.P. (2012). Sources of method bias in social science research and recommendations on how to control it. Annu. Rev. Psychol..

[73] Williams L.J., Anderson S.E. (1994). An alternative approach to method effects by using latent-variable models: Applications in organizational behavior research. J. Appl. Psychol..

[74] Podsakoff P.M., Organ D.W. (1986). Self-reports in organizational research: Problems and prospects. J. Manag..

[75] Maignan I., Ferrell O.C., Ferrell L. (2005). A stakeholder model for implementing social responsibility in marketing. Eur. J. Mark..

[76] Chakraborty A., Jha A. (2019). Corporate social responsibility in marketing: A review of the state-of-the-art literature. J. Soc. Mark..

[77] Gupta R., Sen S. (2013). The effect of evolving resource synergy beliefs on the intentions—Behavior discrepancy in ethical consumption. J. Consum. Psychol..

[78] Zemack-Rugar Y., Rabino R., Cavanaugh L.A., Fitzsimons G.J. (2016). When donating is liberating: The role of product and consumer characteristics in the appeal of cause-related products. J. Consum. Psychol..

[79] Ahmad N., Ullah Z., Ryu H.B., Ariza-Montes A., Han H. (2023). From corporate social responsibility to employee well-being: Navigating the pathway to sustainable healthcare. Psychol. Res. Behav..

[80] Ali M., Islam T., Mahmood K., Ali F.H., Raza B. (2021). Corporate social responsibility and work engagement: Mediating roles of compassion and psychological ownership. Asia-Pac. Soc. Sci. Rev..

[81] Castells M. (1977). The Urban Question: A Marxist Approach.

[82] Lee S.C., Jung S.H., Kim Y.J. (2017). How authenticity evaluation of CSR activities affect brand attitude and usage intention: Focused on the moderating effect of self-construals. Korean J. Commun. Stud..

